# *In Vivo* Emergence of Dual Resistance to Rifampin and Levofloxacin in Osteoarticular *Cutibacterium avidum*

**DOI:** 10.1128/spectrum.03687-22

**Published:** 2023-06-08

**Authors:** Scott A. Cunningham, Christophe Rodriguez, Paul-Louis Woerther, Christophe Menigaux, Thomas Bauer, Jean-Louis Herrmann, Martin Rottman, Anne-Laure Roux, Jean-Louis Gaillard, Robin Patel, Faten El Sayed

**Affiliations:** a Division of Clinical Microbiology, Department of Laboratory Medicine and Pathology, Mayo Clinic, Rochester, Minnesota, USA; b NGS Platform, IMRB, CHU Henri Mondor, Créteil, France; c Institut Mondor de Recherche Biomédicale, Inserm U955, Créteil, France; d APHP, GHU Paris Saclay, Hôpital Ambroise Paré, Orthopedic Surgery Department, Boulogne-Billancourt, France; e APHP, GHU Paris Saclay, Hôpital Raymond Poincaré, Microbiology Department, Garches, France; f Université Paris-Saclay, UVSQ, Inserm, Infection et Inflammation, Montigny-Le-Bretonneux, France; g APHP, GHU Paris Saclay, Hôpital Ambroise Paré, Microbiology Department, Boulogne-Billancourt, France; h Division of Public Health, Infectious Diseases and Occupational Medicine, Mayo Clinic, Rochester, Minnesota, USA; University of Reims Champagne-Ardenne, Biomatériaux et Inflammation en Site Osseux

**Keywords:** *Cutibacterium avidum*, antibiotic resistance, bone and joint infections, *in vivo* emergence of resistance, mechanisms of resistance, osteoarticular infection

## Abstract

Cutibacterium avidum is an emerging causative agent of orthopedic device-related infections (ODRIs). There are no guidelines for the antimicrobial treatment of *C. avidum* ODRI, but oral rifampin is frequently used in combination with a fluoroquinolone following intravenous antibiotics. We describe the *in vivo* emergence of combined resistance to rifampin and levofloxacin in a *C. avidum* strain isolated from a patient with early-onset ODRI treated with debridement, antibiotic treatment, and implant retention (DAIR) using rifampin combined with levofloxacin as the oral treatment. Whole-genome sequencing of *C. avidum* isolates before and after antibiotic exposure confirmed strain identity and identified new mutations in *rpoB* and *gyrA*, leading to amino acid substitutions previously reported to be associated with resistance to rifampin (S446P) and fluoroquinolones (S101L), respectively, in other microbial agents, in the posttherapy isolate. Aside from the molecular insights reported here, this study highlights potential limitations of the combination of oral rifampin and levofloxacin in patients undergoing a DAIR procedure for *C. avidum* ODRI and the potential need to evaluate specific optimal therapy for emerging ODRI pathogens.

**IMPORTANCE** In this study, we report for the first time the *in vivo* emergence of dual resistance to levofloxacin and rifampin in *C. avidum* isolated from a patient who received both antibiotics orally in the setting of a salvage debridement and implant retention of an ODRI. Aside from the molecular insights reported here, this study highlights potential limitations of the combination of oral rifampin and levofloxacin in patients undergoing these surgical procedures and the potential need to evaluate specific optimal therapy for emerging ODRI pathogens.

## INTRODUCTION

Cutibacterium avidum is a skin commensal bacterium increasingly recognized as a cause of orthopedic device-related infection (ODRI) (1–3). Similar to Cutibacterium acnes, this species is susceptible to a wide range of antimicrobial agents, including β-lactams, rifampin, clindamycin, and fluoroquinolones (3, 4). Given its excellent oral bioavailability and *in vitro* activity against *Cutibacterium* species, oral rifampin is frequently used in combination with an oral fluoroquinolone or clindamycin following surgery and intravenous antimicrobial treatment.

Here, we report a case of recurrent *C. avidum* ODRI in a patient treated using DAIR (debridement, antibiotics, and implant retention) with rifampin combined with levofloxacin used as the oral treatment. Whole-genome sequencing of pre- and posttreatment isolates showed *C. avidum* to have developed resistance to both rifampin and levofloxacin involving nonsynonymous missense mutations within *rpoB* and *gyrA*, respectively. This report highlights the risk of failure in patients undergoing a DAIR procedure for *C. avidum* ODRI when using an oral combination of rifampin and levofloxacin.

In July 2019, a 46-year-old woman underwent osteosynthesis material implantation for a fracture of the left humerus. Three weeks later, she was referred by her attending physician to the orthopedic emergency department of Ambroise Paré Hospital for sinus tract formation and skin erythema at the site of the surgical wound. She underwent debridement and implant retention surgery. Five intraoperative tissue samples taken at distinct areas around the implant were collected. *C. avidum* grew from five samples while Staphylococcus epidermidis recovered from two samples (see File S1 in the supplemental material for detailed microbiological growth data). Empirical postoperative intravenous antibiotic treatment with daptomycin (10 mg/kg once a day), cefepime (2 g three times a day), and metronidazole (500 mg three times a day) was administered for 6 days and then switched to oral rifampin (900 mg once a day) and levofloxacin (750 mg once a day). She was discharged with oral rifampin and levofloxacin treatment for 11 more weeks. At 2 weeks after the end of antibiotic treatment, she was readmitted for shoulder pain and wound drainage. Serum C-reactive protein was 59 mg/L and radiological examination showed elevated osteolysis without bone consolidation. She underwent another operation for removal of the osteosynthesis material and implantation of a total reverse shoulder prosthesis. Five intraoperative peri-implant tissue samples were collected, yielding *C. avidum* that, in culture, was resistant to rifampin and levofloxacin (recovered from three samples), Staphylococcus capitis (recovered from two samples), and *C. acnes* (recovered from two samples) (supplemental file S1 for detailed microbiological growth data). Empiric postoperative intravenous antibiotic treatment was switched 1 week after surgery to oral amoxicillin (1 g four times a day), amoxicillin-clavulanic acid (1 g four times a day), and clindamycin (600 mg four times a day) for 11 more weeks. The patient was discharged with a clean wound and no findings of infection. Two years later, the patient appeared to be free from infection without evidence of prosthetic dysfunction.

## RESULTS

### Posttreatment, FMS4815 developed high-level resistance to rifampin and levofloxacin.

In contrast to the pretreatment isolate FMS2275, FMS4815 showed no inhibition zone to both rifampin and levofloxacin when tested by Kirby-Bauer disk diffusion. FMS4815 was otherwise indistinguishable from FMS2275 with respect to colony morphology, hemolytic activity, and growth characteristics on solid media. Using the Bactec FX system, the mutant FMS4815 was detected slightly later than FMS2275 when cultured in Lytic-Ana blood culture bottles (BCBs) with times to detection of 66 and 60 h, respectively. Diameters of inhibition zones and MICs values determined for the two strains are shown in Table 1. FMS4815 was confirmed to have high-level resistance (MIC >32 mg/L) to both rifampin and levofloxacin. Susceptibility to moxifloxacin was also affected, although to a lesser extent than that to levofloxacin, with the MIC value rising from 0.064 mg/L for FMS2275 to 0.75 mg/L for FMS4815 (a 12-fold increase). No changes in MIC values were observed for amoxicillin or clindamycin.

**TABLE 1:** MICs and diameters for pretreatment (FMS2275) and posttreatment (FMS4815) C. avidum isolates^*a*^

Antibiotic	MIC (mg/L)		Diameter (mm)	
	FMS2275	FMS4815	FMS2275	FMS4815
Amoxicillin	0.094	0.094	42	42
Clindamycin	<0.016	<0.016	42	42
Levofloxacin	0.064	>32	38	20
Moxifloxacin	0.064	0.75	45	33
Rifampin	0.002	>32	43	19

a FMS2275, pretreatment *C. avidum* isolate; FMS4815, posttreatment *C. avidum* isolate. MICs were obtained by using the Etest method. Diameters for inhibition zones were obtained by using Kirby-Bauer disk diffusion.

### FMS4815 displays nonsynonymous missense mutations in both *rpoB* and *gyrA*.

FMS2275 and FMS4815 had genome sizes of 2.46 megabases and similar GC contents (63.47 and 63.48% for FMS2275 and FMS4815, respectively). Their core genomes (targeting 56% of the genome) also showed high identity (99.93%), with 16 single nucleotide polymorphisms (SNPs) identified. By comparison, publicly available *C. avidum* genomes differed from each other, on average, by 35,422 SNPs, with a range of 146 to 64,242 SNPs. There was thus strong evidence that FMS4815 was derived from FMS2275.

The 16 SNPs by which FMS2275 and FMS4815 differed were scattered throughout the genome. Among them, five were in coding genes and associated with amino acid substitutions (i.e., nonsynonymous missense mutations) (Table 2). Two mutations were of particular interest. One was within cluster I of the gene encoding DNA-directed RNA polymerase subunit beta (*rpoB*), the target of rifampin (5). The second was within the gene encoding DNA gyrase subunit A (*gyrA*), a topoisomerase targeted by fluoroquinolones (6).

**TABLE 2:** Amino acid changes identified in FMS4815

Gene product	Protein ID	Amino acid change^*a*^
DNA-directed RNA polymerase subunit beta	WP_015582151.1	S446P
Hypothetical protein	WP_004811151.1	L130R
Aminoacyl-tRNA hydrolase	WP_015583161.1	S42A
Alkaline phosphatase family protein	WP_015583500.1	D57A
DNA gyrase subunit A	WP_015583540.1	S101L

a FMS2275→FMS4815 amino acid substitution. The number indicates the position of the amino acid substituted within the polypeptide chain.

The mutation within *rpoB* led to the change of a serine to a proline at position 446 (S446P) (Table 2). Such a mutation has not been reported thus far in *Cutibacterium* species and was not present in the *Cutibacterium* genomes publicly available in the database. It has, however, been reported in S. aureus mutants highly resistant to rifampin (7). The mutation within *gyrA* led to the change of a serine to a leucine at position 101 (S101L) (Table 2), a mutation previously reported in clinical isolates of *C. acnes* and associated with resistance to fluoroquinolones (8).

## DISCUSSION

Here, the *in vivo* emergence of dual resistance to levofloxacin and rifampin in *C. avidum* isolated from a patient who received both antibiotics orally in the setting of a DAIR procedure is reported for the first time. The isolation of *C. avidum* resistant to levofloxacin, but still susceptible to rifampin, has been reported in one case of *C. avidum* ODRI treated with levofloxacin and rifampin (1), but there has been no report of rifampin-resistant *C. avidum* from ODRI.

Two distinct mutations were associated with observed double resistance, one affecting *rpoB* and the other affecting *gyrA*. The mutations were nonsynonymous missense mutations leading to amino acid substitutions in both cases. The mutation within *rpoB* caused a S446P substitution, a signature not yet reported in *Cutibacterium* species but previously described at an equivalent position (S464P) in S. aureus mutants expressing high-level rifampin resistance, as in our case (7).

The mutation within *gyrA* causes a S101L substitution located within the quinolone resistance-determining region, and alters the binding site of fluoroquinolones. This substitution has been reported in association with fluoroquinolone resistance in *C. acnes*. The same substitution at equivalent positions in E. coli (S83L) and S. aureus (S84L) resistant to fluoroquinolone were also reported (8–11). An S101L substitution is the most common change found in fluoroquinolone-resistant *C. acnes* mutants selected *in vitro* and *in vivo* (9, 10). These mutants show not only high-level resistance to levofloxacin but also a decrease in moxifloxacin susceptibility. This is consistent with the findings reported here, with MIC values for the mutant strain increasing ranging from 0.002 to >32 mg/L for levofloxacin (>16,000-fold increase) and from 0.064 to 0.75 mg/L (12-fold increase) for moxifloxacin.

The occurrence of a double chromosomal mutation, each associated with high-level resistance involving families of different antibiotics, is concerning. In the case reported above, exposure of *C. avidum* to levofloxacin may have favored the appearance of the mutation targeting *rpoB*. Indeed, *in vitro* studies in S. aureus show that the emergence of rifampin-resistant mutants by mutation of *rpoB* is greater in the presence than in the absence of ciprofloxacin (12). Any molecule belonging to this family is potentially capable of exerting such a “mutagenic” effect due to replication errors induced by fluoroquinolones, and this phenomenon could have occurred in the case described with levofloxacin. However, this is hypothetical. Indeed, fluoroquinolone-induced mutations are linked to disruptions in DNA fork progression and replication repair and are typically insertions or deletions. Here, the sequence changes observed in the genome of the posttreatment isolate were point mutations.

The price to pay for a mutation also merits consideration when it involves mutations that affect genes, such as *gyrA* and *rpoB*, involved in major physiological processes. From a general point of view, mutations of *rpoB*, and to a lesser extent *gyrA*, are “costly” and can be associated with a loss of fitness (13). However, the effect varies depending on the specific mutations and the genetic background in which they occur (bacterial species, strain) (13). In the case described above, the growth rate of the double mutant was slightly slower, as determined by the time to detection in the Bactec FX system, than for the susceptible strain. This modest impact in the context of high levels of resistance is consistent with the clinical observation, since the resistant mutant developed over a few months, replaced the initial susceptible strain, and caused a clinical, biological, and radiological infectious relapse (in association with a strain of *C. acnes* and one of *S. capitis*, found in lesser quantities).

We cannot exclude the possibility that *C. avidum*, or even our specific strain, has a particular ability to develop and accumulate such resistance without a significant fitness cost due to a genetic background at the origin of compensatory processes. It is indeed notable that the same antibiotic treatment that led to the emergence of the rifampin- and levofloxacin-resistant double mutant of *C. avidum* allowed eradication of S. epidermidis initially found in association with *C. avidum*. In addition, *C. acnes* and *S. capitis* found to be associated with the rifampin- and levofloxacin-resistant double mutant of *C. avidum* posttreatment showed no resistance to rifampin or levofloxacin. However, these species were found at a lower abundance than *C. avidum*, and the nonemergence of resistant mutants could be due to an insufficient bacterial load to observe such mutants, even in the case of single rifampin- or levofloxacin-resistant mutants.

This case report echoes current doubts about the possibility of retaining implants in cases of ODRI caused by *C. avidum*. In a recent series of 13 *C. avidum* PJI cases, treatment failed in all but one of the six patients who underwent debridement-retention of the prosthesis, necessitating two-stage revision of the arthroplasty, with a good clinical outcome (1). In another series of 14 cases of “chronic” *C. avidum* prosthetic hip infection treated by one-stage exchange, only one relapse occurred. Maintaining the implant therefore appears to be associated with a high risk of failure in cases of *C. avidum* ODRI, either by maintaining a bacterial load that is too high and/or protection of bacteria in the form of a biofilm (2). Thus, as stated by Achermann et al. (1), one- or two-stage revision should be favored over debridement-retention of the implant for a successful treatment outcome in *C. avidum* ODRI.

Another question concerns the choice of oral antibiotics. This question is more difficult to address because the treatments used have been extremely heterogeneous in case series of *C. avidum* ODRI (1, 2, 14). Further, while it is tempting to rely on the extensive data concerning *C. acnes*, it is not certain these data can be applied to *C. avidum*, because of the different pathogenicity profiles (supposed higher virulence of *C. avidum*). There is therefore currently no consensus concerning the choice of molecules for antibiotic treatment of C. *avidum* ODRI, in particular, antibiotics that can be administered orally.

The choice is relatively broad because *C. avidum* is naturally susceptible to several oral antibiotics commonly used in orthopedic surgery, including amoxicillin, clindamycin, rifampin, and fluoroquinolones, such as levofloxacin. Acquired resistance to these molecules is rare, apart from resistance to clindamycin (4), the prevalence of which can exceed 30% in some centers (2). As reviewed by Corvec (3), most experts recommend combinations, including rifampin, given the very high activity of rifampin against *C. avidum* and its excellent bone distribution and diffusion through biofilms. Rifampin is also useful when staphylococci are associated with *C. avidum*, as in our case report. In most oral treatments reported in the literature, antimicrobial agents administered concomitantly with rifampin have been amoxicillin, clindamycin, and a fluoroquinolone, most often levofloxacin, but sometimes moxifloxacin (1, 2).

Data obtained *in vitro* with *C. acnes*, however, should be noted; penicillin G, clindamycin, and levofloxacin were unable to prevent the emergence of rifampin-resistant mutants when a high bacterial inoculum was used (10^8^ CFU of *C. acnes*) (15). Given potential similarity with *C. avidum*, this implies a risk of failure from emergence of a rifampin-resistant mutants in the presence of a heavy bacterial load, whether the associated molecule is penicillin G (and probably amoxicillin), clindamycin, or levofloxacin. Similar results have been shown *in vitro* with daptomycin, highlighting the difficulty of obtaining an optimal combination with rifampin (15).

In conclusion, this case report supports changing the implant in the management of ODRI due to *C. avidum*. The emergence of a mutant resistant to rifampin and levofloxacin under a treatment combining these two molecules calls for caution concerning the use of this association and, pending additional data, administration of rifampin with other fluoroquinolones (e.g., moxifloxacin). As recently highlighted by several authors, there is a need to further define the optimal surgical and medical treatment of *C. avidum* ODRIs.

## MATERIALS AND METHODS

### Routine bacteriological procedures.

Procedures for routine peri-implant tissue culture were performed as previously described (16). Briefly, tissue samples were homogenized in sterile water using a bead mill, and homogenates were inoculated onto (i) solid media and incubated for 5 days (Columbia 5% sheep blood agar under aerobic and anaerobic conditions and chocolate agar in a 5% CO_2_-enriched atmosphere), (ii) aerobic BCBs (BD Bactec PedsPlus; Becton Dickinson Diagnostics, Sparks, MD) and incubated for 7 days, and (iii) anaerobic BCBs (BD Bactec Lytic/10 Anaerobic/F, Lytic-Ana) and incubated for 14 days. Aerobic and anaerobic BCBs were monitored in a Bactec FX instrument (Becton Dickinson Diagnostics) and subcultured only when a positive signal was indicated. Bacterial isolates were identified by matrix-assisted laser desorption ionization–time of flight (MALDI-TOF) mass spectrometry using a Microflex LT instrument and CE-labeled IVD Biotyper software (v4.2.28; Bruker Daltonik, Wissenbourg, France). Several *C. avidum* colonies were randomly selected from each positive tissue sample (as one isolate) for (i) antibiotic susceptibility testing by Kirby-Bauer disk diffusion (according to French CASFM recommendations) and (ii) cryopreservation at −80°C.

### Selection of pre- and posttreatment isolates for further analysis.

Cryopreserved *C. avidum* isolates from the initial episode of infection (“pretreatment”) and the second episode (“posttreatment”) were revivified by culture on 5% Columbia sheep blood agar under anaerobic conditions. Isolates were then reassessed by MALDI-TOF mass spectrometry and Kirby-Bauer disk diffusion, as described above. Antibiotic susceptibility testing was performed in triplicate on each *C. avidum* isolate. All pretreatment isolates were susceptible to rifampin and levofloxacin, whereas all posttreatment isolates were resistant (no inhibition zone) to both agents. One pretreatment isolate (FMS2275) and one posttreatment isolate (FMS4815) were randomly selected and cryopreserved for further analysis.

### Colonial aspect and growth characteristics.

FMS2275 and FMS4815 were cultured for 5 days on 5% Columbia sheep blood agar under anaerobic conditions and chocolate agar in a 5% CO_2_-enriched atmosphere. Evaluation for colonies was evaluated daily and hemolysis after 48 h of incubation on blood agar. Growth kinetics were evaluated using the Bactec FX system. Lytic-Ana BCBs were inoculated with 100 CFU (1 mL in saline) and loaded into the Bactec FX instrument. Growth was monitored for 14 days or until positivity. Positive vials were subcultured on solid media (aerobic and anaerobic conditions), and colonies were identified by MALDI-TOF mass spectrometry. Experiments were performed in duplicate.

### Determination of MIC values.

Amoxicillin, rifampin, clindamycin, levofloxacin, and moxifloxacin MICs were determined using Etest strips (bioMérieux, France). Experiments were performed in triplicate.

### Genomic study.

For whole-genome sequencing, cultured isolates were diluted to 1 McFarland in sterile water, pre-extracted by bead beating, and extracted using a DSP DNA Midi kit on a QIAsymphony instrument (Qiagen), as described previously (17). Extracts were prepared using a Nextera XT kit (Illumina) and pair-end sequenced (2 × 150 bp) using a NextSeq 500/550 High-Output kit v2.5 (300 cycles) on a NextSeq500 instrument (Illumina). Adapter and index sequences were removed with Atropos version 1.1.31 (18). Clipped read files were *de novo* assembled with SPAdes and polished with Unicycler, with resultant assemblies annotated by using the RAST tool kit in the PATRIC workbench (19). Draft genomes of both FMS2275 and FMS4815 are publicly available on NCBI GenBank under accession numbers SAMN23427968 and SAMN23427969, respectively. FASTA assembly files were quality checked with QUAST (20) and imported into SeqSphere+ v7.2.6 (Ridom, Münster, Germany) for core genome multilocus sequence analysis (cgMLST) (21). First, an *ad hoc* cgMLST scheme was created in SeqSphere+ using the *C. avidum* 44067 reference genome (NC_021064.1) as the seed genome. A total of 22 genomes available in the National Center for Biotechnology Information (NCBI) were BLAST queried for potential core genome targets using default filter settings within SeqSphere+. The BLAST query identified 1,300 allelic targets compromising the core genome (cgMLST targets). A project pipeline was created within SeqSphere+, and assembled genomes for the two clinical isolates and assemblies from the 23 publicly deposited isolates within the NCBI database were processed. SeqSphere+ was used to perform reference genome/cgMLST target mapping against the *C. avidum* 44067 reference genome for each of the assembly files and create a cgMLST allelic comparison table at the conclusion of the pipeline. SNPs between core genomes were derived from the comparison table data. In parallel, genome-wide SNP comparisons were made using Snippy (https://github.com/tseemann/snippy). Alleles associated with observed antibiotic resistance (*gyrA* and *rpoB*) were excised from annotated genomes for both clinical isolates. *gyrA* and *rpoB* sequences for each of the isolates were aligned to homolog sequences in Escherichia coli K-12 (NZ_CP010441.1), Mycobacterium tuberculosis H37Rv (NC_000962.3), Staphylococcus aureus NCTC 8325 (NC_007795.1), Cutibacterium acnes HL096PA1 (NC_021085), and Cutibacterium avidum 44067 (NC_021064.1) using the CLC Genomics Workbench (v21; Qiagen, Redwood City, CA).

### Data availability.

Draft genome of both FMS2275 and FMS4815 are publicly available on NCBI GenBank under accession numbers SAMN23427968 and SAMN23427969, respectively.
