# Supplementary material for: In Vivo Emergence of Dual Resistance to Rifampin and Levofloxacin in Osteoarticular Cutibacterium avidum
Source: Microbiol Spectr. 2023 Jun 8;11(4):e03687-22. doi: 10.1128/spectrum.03687-22 (PMC10434181; doi:10.1128/spectrum.03687-22)
Supplement: Supplemental file 1 — Table S1. Download spectrum.03687-22-s0001.docx, DOCX file, 0.01 MB [file spectrum.03687-22-s0001.docx]

**S1. Detailed microbiological growth data of the five peri-implant intraoperative tissue samples collected on the first and the second episode of infection**

| Samples | BCB Aerobic^1^ | BCB Anaerobic^2^ | Chocolate agar ^3^ | Anaerobic blood agar ^4^ | Aerobic blood agar ^5^ |  |
| --- | --- | --- | --- | --- | --- | --- |
| First episode |  |  |  |  |  |  |
| 1 | *S.epidermidis* (20) | *C. avidum* (52) | *C. avidum* | *C. avidum* | Negative |  |
| 2 | *C. avidum* (70) | *S. epidermidis* (18) | *C. avidum* | *C. avidum* | Negative |  |
| 3 | *C. avidum* (138) | *C. avidum* (60) | *C. avidum* | *C. avidum* | Negative |  |
| 4 | *C. avidum* (59) | *C. avidum* (43) | *C. avidum* | *C. avidum* | Negative |  |
| 5 | *C. avidum* (87) | *C. avidum* (54) | *C. avidum* | *C. avidum* | Negative |  |
| Second episode | |  |  |  |  |  |
| 1 | *C. avidum* (150) | *C. avidum* (69) | *C. avidum* | *C. acnes* | Negative |  |
| 2 | Negative | *C. avidum* (97) | Negative | Negative | Negative |  |
| 3 | Negative | *S. capitis*(43) | Negative | *C. acnes* | Negative |  |
| 4 | Negative | *C. avidum* ; *S. capitis* (78) | *S. capitis* | Negative | Negative |  |
| 5 | Negative | Negative | Negative | Negative | Negative |  |

^1^Aerobic blood culture bottles (BCB) (BD Bactec PedsPlus, Becton Dickinson Diagnostics, Sparks, MD) were incubated for 7 days or until positivity. Time to detection for each species is indicated in hours between brackets.

^2^Anaerobic blood culture bottles (BCB) (BD Bactec Lytic /10 Anaerobic/F; Lytic-Ana) were incubated for 14 days or until positivity. Time to detection for each species is indicated in hours between brackets.

^3^ All *C. avidum* colonies were observed at day 5 of incubation on a chocolate agar in a 5% CO2-enriched atmosphere. *S. capitis* colonies were observed after 48 hours of incubation.

^4^ All colonies were observed at day 5 of incubation on a Columbia 5% sheep blood agar under anaerobic conditions.

^5^ Columbia 5% sheep blood agar plates were incubated 48 hours under anaerobic conditions.
